# Some Selected Skin Cases

**Published:** 1911-08-12

**Authors:** G. Norman Meachen

**Affiliations:** Physician to the Blackfriars Skin Hospital; Physician-in-Charge of Skin Department, Prince of Wales's Hospital; Dermatologist to the Foundling Hospital.


### Hospital Clinics.

### SOME SELECTED SKIN CASES.

By G. NORMAN MEACHEN, M.D., B.S., M.R.C.P., Physician to the Blackfriars Skin Hospital; Physician-in-Charge of Skin Department, Prince of Wales's Hospital; Dermatologist to the Foundling Hospital.

(Delivered at the North-East London Post-Graduate College.)

The first case I have to show you this afternoon is this little boy of three with well-marked congenital xerodermia. The mother states that "immediately after birth the skin began to peel like after scarlet fever," and the integument has remained harsh and dry, more or less, ever since. One other child in the family is affected in a similar way, and the father is stated to have a "dry skin every seven years." As is usual in these cases, the condition is most marked upon the shins and upon the extensor surfaces of the limbs and trunk. In an exaggerated degree the condition is known as ichthyosis, when scaliness is a more obvious feature. The general health never suffers, but the "dirty" looking state of the skin is apt to attract notice, and, indeed, school teachers have been known to send children thus affected home to be washed. Much can be done in the way of treatment for xerodermia, glycerine of starch being a favourite application, 2 drachms in equal parts of the B.P. ung. glyc. plumbi. subacet. and lanoline, to which a few grains of resorcin may be added with some advantage.

### Granuloma Annulare.

This little girl of five has a rare condition, granuloma annulare, upon her right fore-arm near the wrist. The lesion began as a "pimple" or "red spot" three months ago, and it has gradually spread to its present size. When I first saw her there was a circinate lesion, whitish-red in colour, with a peculiar, lumpy, nodular margin, quite painless, and unaccompanied with itching. There was no history of tuberculosis in the family, such as is frequently met with in this affection. The name of the disease was given to it by the late Dr. Radcliff Crocker, and it is likely to stick, in spite of the fact that, histologically, the condition does not present the features common to the granulomata as a class, the hypodermic inflammation being generally observed around the blood-vessels. Some of you may remember the case of a young man from the country whom I showed here last year with a similar condition upon the knuckles. That patient is now nearly well as the result of the application of an ointment of salicylic acid, though sometimes, as Dr. Graham Little has pointed out in his monograph upon the subject published in vol. 1 of the Proceedings of the Royal Society of Medicine (Dermatological Section), the lesions will disappear after simply bandaging the part with firm pressure. Clinically, the condition may be mistaken for tinea circinata, or an erythema, but neither of these present the characteristic, infiltrated nodules at the margin of the growth.

### Lupus.

Here is a youth of seventeen, much undersized, whom I referred to my colleague, Mr. Carson, some two years ago for laryngeal trouble complicating his lupus, from which you will see he is suffering extensively. The patches upon the left arm were the first to appear, and then the nose began to be attacked, and patches appeared upon the sides of the neck, the forehead, and now the scalp presents a large area with scabbing and cicatricial atrophy. I understand the laryngitis is considerably better, though the voice is still husky, and he has been receiving pretty regular injections of tuberculin from Dr. McDonald, without, I fear, much influence upon the lupus. There is a small lupoid area just developed in front of the hard palate. I think that the *x*-rays will be of some service as far as the nose is concerned. (Replying to a question as to whether such a case as this was not a danger to the community, the lecturer thought that the risks were no greater than in phthisis, but rather less, seeing that the tubercle bacillus was confined to the deeper parts of the lesions.)

### Lupus Erythematosus.

As a contrast case may be taken this Jewish woman, aged thirty, with ul-erythema (lupus erythematosus) of her scalp. The condition has existed for upwards of four years, and it has been accompanied with much burning pain and neuralgia. You see that the central part of the affected area is quite smooth and cicatricial, but there is no scabbing and no exudation, as in the previous case. Moreover, the edges, where the disease is active, are merely reddened, slightly scaly, and raised, with here and there some enlarged follicles. The baldness will be permanent, as the hair-papillæ are destroyed by the atrophic process. We shall give her quinine internally, and locally we may paint the spreading edges cautiously with the liq. sodii ethylatis, or, if this produce too strong a reaction, with equal parts of powdered camphor and pure phenol. Some of these cases have done well with cataphoresis with zinc salts. This case is remarkable in that the scalp alone is affected. Beyond a small capillary nævus upon her nose, and some general hirsuties, she has no other lesions upon the face, and this is not at all common. I showed a patient, a widow, aged fifty-four, two years ago before the Dermatological Section of the Royal Society of Medicine, in which the scalp was extensively affected, but she also had one small lesion upon the nose. In this latter case there was a strong family history of tuberculosis, as she had lost no fewer than eight children with varying manifestations of that disease. I cannot trace any similar history in the case now before us.

Here is a young man, aged sixteen, with occupation-dermatitis, involving both the fore-arms and hands. He has been working at a cabinet-maker's for some time past and has been using a polish containing bichromate of potash. The condition began six months ago, when he was medically treated, and with rest from work it got practically well. He returned to work two days back, and now, he says, he is as bad as he was before. The question is often raised as to the difference between a dermatitis caused by some irritant applied from without and a true eczema, and I would say that, in a large majority of cases, there is absolutely no difference, either clinically or pathologically. In both there may be a true "catarrhal inflammation" of the skin, with weeping, papule formation, and even secondary coccal infection. It is more usual to speak of the cases directly connected with the handling of some irritating substance, whether this be alkalies, dyes, sugar, certain plants, or photographic materials—to name only a few typical ones —as being a trade or an occupation-dermatitis, for we are tending gradually to use the word "eczema" less and less in dermatological nomenclature. Such individuals are often terribly handicapped because they cannot stay away from their work, so that the treatment does not get a fair chance to cure. The wearing of gloves, too, is not always possible in certain occupations. It is sometimes a puzzle to know why a person who has been engaged in a certain occupation for a long period, using, perhaps, some irritating substance daily with impunity, suddenly becomes affected with a dermatitis, and I can only explain this fact by assuming that his powers of resistance have been lowered in some way, so that the eruption breaks out. I have satisfied myself that this is so in certain cases of dermatitis in gardeners. To recommend staying away from work is, alas! too often a counsel of perfection for hospital out-patients, but in severe cases the removal of the exciting cause becomes a necessity. I have prescribed a simple calamine lotion for this youth, with an ointment of zinc oxide and lanoline, to which we may add a little ichthyol at his next visit.

This boy of ten, kindly sent to me by Dr. Tresilian, of Enfield, has an interesting condition of skin. About a fortnight ago an eruption is stated to have appeared upon the side of the chest and abdomen, and to have spread gradually upwards and downwards. It was rather bright-red in colour, with slight irritation and some scaliness. As you see him now it would be distinctly difficult to say off-hand from what cutaneous affection he is suffering, but I think, from a careful examination of the facts, that he is recovering from pityriasis rosea. This is not a very common disease, but is seen, especially in childhood and early adult life as a circinate, pinkish, scaly eruption, generally appearing first upon the body and spreading slowly to the limbs, rarely involving the face. It is a little like psoriasis, but the lesions are never so scaly nor do they last so long. The affection more often simulates tinea circinata, and, in fact, the Vienna school taught that pityriasis rosea was, in reality, a disseminated ringworm, but this view has never been accepted in this country, and, moreover, no fungus can be obtained on scraping the lesions. This latter point distinguishes it absolutely from tinea circinata. This boy is now in the "clearing-up stage" of the disease, in which a superficial coalescence, with slight dirty scaliness, of the lesions is often observed. Happily, the condition soon disappears, and a mild tar lotion, combined with the inunction of an ointment containing a little liq. carb. deterg. or a few minims of cyllin will cure it.

My last patient is this woman, aged thirty-eight, with lichen planus. I am showing her to you on account of the incipient warty or verrucose stage of the disease which is just appearing upon the shins. If you saw no other lesions besides these, it might be almost impossible to distinguish between lichen planus and a psoriasis undergoing treatment. Two diagnostic points would, I think, prevent such confusion. In the first place, in the near neighbourhood of these patches may be seen, on careful examination with a hand-lens, one or two of the typical, small, flattened, lesions of lichen, and these may also be seen in those situations favoured by the eruption, such as upon the inner sides of the knees and upon the flexor aspects of the wrists. Secondly, the patches are not in the least scaly, but merely thickened or slightly infiltrated. Colour *per se* does not help us much at this stage, though at the first appearance of the eruption the dull red, or even bluish tint of some of the plane lesions is sufficiently diagnostic. In the early acute stages of the disease it is quite common to find lesions upon the buccal mucous membrane, either as whitish dots, or, as Brocq has so aptly described, as streaks comparable with the appearance produced by touching the surface with a stick of silver nitrate. This patient does not show anything inside the mouth. Her general health is good, and in her case we could not find anything in her history to account for the eruption in the way of some nervous shock, which is such a common antecedent of lichen planus. She is now under gradually increasing doses of Fowler's solution, and the disease is showing signs of disappearing. If arsenic is not well borne, then mercury is the next best drug. Local irritation may be quelled by bathing with hot cyllin lotion, and an ointment of 5 or 6 grains of salicylic acid with 20 minims of cyllin to the ounce may be applied to the lesions.

